# Lea’s Series of Pocket Text-Books—Anatomy

**Published:** 1903-10

**Authors:** 


LEA'S SERIES OF POCKET TEXT-BOOKS—ANATOMY.—A Manual for Students and Practitioners. By William H. Rockwell, Jr., M. D., formerly Assistant Demonstrator of Anatomy in the College of Physicians and Surgeons, Columbia University, New York. Edited by Bern B. Gallaudet, M. D., Demonstrator of Anatomy, etc., in the College of Physicians and Surgeons, New York. Illustrated with seventy engravings. Lea Bros. & Co., Philadelphia and New York. 1903.

In preparing this manual the author has closely followed *Gray's Anatomy,* both in order and in description. It thus can be conveniently studied in conjunction with that recognized authority.

Dr. Rockwell explains that his object in offering this work to the profession is not to replace any of the larger works on anatomy, but rather to present the subject in a briefer and more readily accessible form.

In every respect the work is all that could be desired.

